# Evaluation of Macro- and Microelement Levels in Black Tea in View of Its Geographical Origin

**DOI:** 10.1007/s12011-016-0849-2

**Published:** 2016-09-16

**Authors:** Justyna Brzezicha-Cirocka, Małgorzata Grembecka, Tomasz Ciesielski, Trond Peder Flaten, Piotr Szefer

**Affiliations:** 1 Department of Food Sciences, Medical University of Gdańsk, Al. Gen. J. Hallera 107, 80-416 Gdańsk, PL Poland; 2 Department of Biology, Norwegian University of Science and Technology, NO-7491 Trondheim, Norway; 3 Department of Chemistry, Norwegian University of Science and Technology, NO-7491 Trondheim, Norway

**Keywords:** Factor analysis, Cluster analysis, RDA, PTWI, Black tea, FAAS

## Abstract

The aim of this study was to evaluate the elemental composition of black tea samples and their infusions in view of their geographical origin. In total, 14 elements were analyzed, 13 (Ca, K, Mg, Na, Mn, Fe, Zn, Cu, Cr, Ni, Co, Cd, and Pb) by flame atomic absorption spectrometry, and P by UV-Vis spectrometry, after mineralization of samples. It was found that K was the most abundant macroelement in the analyzed samples, whereas among microelements, the highest concentration was found for Mn. Based on the obtained data, the percentage of elements leached into the infusions as well as the daily elemental intake from tea were calculated. The daily intake from tea was compared to the recommended daily allowances (RDAs), and the highest percentages of the RDAs were found for Mn (15 %) and Co (10 %). To study the relations between elemental composition and country of origin of samples, factor analysis and cluster analysis were applied. These multivariate techniques proved to be efficient tools able to differentiate samples according to their provenance as well as plantation within the common regions.

## Introduction

According to the Food and Agriculture Organization of the United Nations Statistics Division (FAOSTAT), total tea production was estimated at 5.3 million tons in 2011, which makes it the most commonly consumed beverage around the world. Tea production in Europe reaches 513,000 tons per year [1]. The main world tea producers are China and India (1.4 and 0.92 million tons, respectively) [2], whereas in Europe, the Russian Federation and the UK (184,000 tons and 129,000 tons, respectively) are the leading producers [1]. The countries with the highest rate of tea consumption include Paraguay and Afghanistan, 7.93 and 4.55 kg/year/person, respectively [2]. According to FAOSTAT data, Malta has the largest tea consumption per capita in Europe amounting to 2.24 kg/year [1], followed by the UK and Ireland, whereas Poland is in seventh place with 0.92 kg/year [1]. According to Polish literature, the average Polish resident consumes 2–3 cups of tea per day, and 20 % of the population consumes 4–5 cups per day [3]. Many studies have reported positive effects of tea consumption on human health, such as cancer prevention, diabetes management by reducing glucose and cholesterol levels in blood, and improved immune defense [4]. Most of the tea world consumption is black tea (80 %), whereas the remaining 20 % belong to green, oolong, red, and yellow types of this beverage. The price of tea products as well as consumer interests are usually connected with the certified geographical origin [5]. Therefore, it is important to have tools that are able to ensure good quality of products, which is usually associated with their geographical provenance. There are several studies of authentication of tea origin, using multivariate chemometric techniques, such as principal component analysis (PCA), factor analysis (FA), cluster analysis (CA), or linear discriminant analysis (LDA) [5]. These techniques have been applied to elemental and organic composition data, obtained using various analytical techniques including HPLC [6–8], GC-MS [9–11], ^1^HNMR [12], FT-NIRS [13], ICP-MS [5, 14–16], and FAAS [17–20]. Our previous studies have showed that FA and CA are efficient tools of green, fruit, and Pu-erh tea diversification [19, 20]. These two multivariate techniques applied to green tea data enabled differentiation of samples according to their provenance [19]. Moreover, it was also possible to diversify Pu-erh tea according to its type of confection, whereas fruit teas were differentiated according to their type [20]. Such information is of great importance to consumers, who expect that price is an equivalent of good quality products.

Therefore, in this study, we aimed to verify the geographical origin of commercialized black tea samples applying factor analysis (FA) and cluster analysis (CA) to their elemental composition. These techniques were found useful to determine the country of origin of the tea as well as its provenance within a single country. In addition, the percentage of elements leached into the tea infusions was determined, and the daily elemental intake from tea was calculated and compared to the recommended daily allowances (RDAs). For Cd and Pb, the intake from tea was compared to the provisional tolerable weekly intake (PTWI).

## Materials and Methods

### Samples

The analyzed tea samples (loose form and tea bags) were purchased from markets and tea shops (original tea) in Poland, and analyzed for their content of 14 elements: K, Na, Ca, Mg, Mn, Zn, Cu, Fe, P, Co, Ni, Cr, Cd, and Pb. In total, 118 types of black tea from different producers were tested, i.e., 708 analytical samples of black tea leaves and their infusions were prepared. Of the 118 tea types analyzed, 43 were purchased in tea shops (12 Chinese, 17 Indian, 10 Ceylon, and 4 Kenyan teas), and 75 in markets (loose form and tea bags) (Table 1).

**Table 1:** Characteristics of the analyzed products

No.	Name of tea	Producer	Country/producer declaration	Confection
Original tea				
1.	Ceylon FBOPF “Malatiyana”	Maraska	Ceylon	Loose
2.	Ceylon OP “Lumbini”	Maraska	Ceylon	Loose
3.	Ceylon “Kendy”	Maraska	Ceylon	Loose
4.	Ceylon UVA OPI “Ivy Hills”	Maraska	Ceylon	Loose
5.	Ceylon Dimbula	Five o’clock	Ceylon	Loose
6.	Ceylon Raigama Korales	Five o’clock	Ceylon	Loose
7.	Ceylon Pothotuwa	Five o’clock	Ceylon	Loose
8.	Ceylon Sithaka FBOPFEXS W	Five o’clock	Ceylon	Loose
9.	Ceylon Earl Grey	Five o’clock	Ceylon	Loose
10.	Ceylon High Grown	Time to tea	Ceylon	Loose
11.	Yunnan Golden OP	Maraska	China	Loose
12.	Golden Monkey	Maraska	China	Loose
13.	China OP Keemun	Maraska	China	Loose
14.	Yunnan Special Black	Five o’clock	China	Loose
15.	China Keemun Mao Feng	Five o’clock	China	Loose
16.	Golden Yunnan^a^	Five o’clock	China	Loose
17.	Lapsang Souchong^a^	Five o’clock	China	Loose
18.	China Black Golden Monkey	Five o’clock	China	Loose
19.	Lapsang Souchong^a^	Time to tea	China	Loose
20.	China Keemun Congu	Time to tea	China	Loose
21.	Yunnan Black Premium	Time to tea	China	Loose
22.	Golden Yunnan^a^	Time to tea	China	Loose
23.	Darjeeling FTGFOPI “Himalaya”	Maraska	India	Loose
24.	Assam TGFOPI “Dikom”	Maraska	India	Loose
25.	Assam TGFOP “Ambaguri”	Maraska	India	Loose
26.	Assam Jamguri FTGFOP1	Five o’clock	India	Loose
27.	Assam Dagapur	Five o’clock	India	Loose
28.	Darjeeling Castleton 2011 WP	Five o’clock	India	Loose
29.	Assam Satishpur TGFOP W	Five o’clock	India	Loose
30.	Assam Marangi FTGFOP1	Five o’clock	India	Loose
31.	Darjeeling Thurbo FTGFOP	Five o’clock	India	Loose
32.	Assam Halmari GTGFOP CL W	Five o ‘clock	India	Loose
33.	Darjeeling Margarets Hope	Five o’clock	India	Loose
34.	Assam Dikom	Time to tea	India	Loose
35.	Assam Sec. Flush	Time to tea	India	Loose
36.	Assam Dekorai	Time to tea	India	Loose
37.	Darjeeling Gielle	Time to tea	India	Loose
38.	Darjeeling First Flush	Time to tea	India	Loose
39.	Darjeeling Sec. Flush	Time to tea	India	Loose
40.	Kenia GFOP “Milima	Maraska	Kenia	Loose
41.	Kenia TGFOP Golden Tipped	Maraska	Kenia	Loose
42.	Kenia Marinyn	Five o’clock	Kenia	Loose
43.	Ruanda Rukeri	Five o’clock	Kenia	Loose
Marketed tea				
1.	English Breakfast^a^	Ahmad Tea	Ceylon	Loose
2.	Ceylon Tea	Ahmad Tea	Ceylon	Loose
3.	Earl Grey Tea^a^	Ahmad Tea	Ceylon	Loose
4.	Earl Grey^a^	Ahmad Tea	Ceylon	Bags
5.	English Breakfast^a^	Ahmad Tea	Ceylon	Bags
6.	English No.1	Ahmad Tea	Ceylon	Bags
7.	Assam^a^	Ahmad Tea	Ceylon	Bags
8.	Ceylon	Ahmad Tea	Ceylon	Bags
9.	English V.I.P Tea	Brizton	Ceylon	Bags
10.	English Royal Tea^a^	Chelton Tea Collection	Ceylon	Loose
11.	Scottish Breakfast	Chelton Tea Collection	Ceylon	Loose
12.	English Royal Tea^a^	Chelton Tea Collection	Ceylon	Bags
13.	Ceylon Supreme Tea	Dilmah	Ceylon	Loose
14.	Earl Grey Tea^a^	Dilmah	Ceylon	Loose
15.	Meda Watte	Dilmah	Ceylon	Loose
16.	Ran Watte	Dilmah	Ceylon	Loose
17.	Uda Watte	Dilmah	Ceylon	Loose
18.	English Breakfast Tea	Dilmah	Ceylon	Loose
19.	English Afternoon Tea	Dilmah	Ceylon	Bags
20.	Premium Tea	Dilmah	Ceylon	Bags
21.	Ceylon Gold	Dilmah	Ceylon	Bags
22.	Perfect Ceylon Tea	Dilmah	Ceylon	Bags
23.	Elegant Earl Grey	Dilmah	Ceylon	Bags
24.	Ceylon OP	Drury	Ceylon	Loose
25.	Royal Ceylan	Lipton	Ceylon	Loose
26.	Yellow Label Tea^a^	Lipton	Ceylon	Bags
27.	Gold Tea Black	Lipton	Ceylon	Bags
28.	Mild Ceylon	Lipton	Ceylon	Bags
29.	Earl Grey^a^	Sir Roger	Ceylon	Bags
30.	Ceylon Gold	Sir William’s	Ceylon	Bags
31.	Black Tea Ceylon	Sir William’s	Ceylon	Bags
32.	Super Pekoe	Tarlton	Ceylon	Loose
33.	Ceylon Orange Pekoe Tea	Twinings	Ceylon	Bags
34.	Yunnan	Loyd Tea	China	Loose
35.	Prince of Wales	Twinings	China	Bags
36.	Black Tea^a^	Yunnan	China	Loose
37.	Darjeeling^a^	Ahmad Tea	India	Loose
38.	Darjeeling^a^	Ahmad Tea	India	Loose
39.	Assam^a^	Ahmad Tea	India	Loose
40.	Darjeeling^a^	Darvilles of Windsor	India	Loose
41.	Royalty Assam	Darvilles of Windsor	India	Loose
42.	Maharajah Reseve Assam	Dilmah	India	Bag
43.	Rich Assam	Lipton	India	Bags
44.	Darjeeling^a^	Premier’s Tea Limited	India	Loose
45.	Earl Grey^a^	Premier’s Tea Limited	India	Loose
46.	Darjeeling SFTGFOP1	Rich Mont	India	Loose
47.	Earl Grey^a^	Sir William’s	India	Bags
48.	Intensive Tea	Tetley	India	Bags
49.	Darjeeling Tea^a^	Twinings	India	Loose
50.	Darjeeling Tea^a^	Twinings	India	Bags
51.	English Tea No.1	Ahmad Tea	–	Loose
52.	English Breakfast^a^	Time to tea	–	Loose
53.	Earl Grey^a^	Time to tea	–	Loose
54.	English Breakfast^a^	Darvilles of Windsor	–	Loose
55.	Earl Grey^a^	Darvilles of Windsor	–	Loose
56.	Earl Grey^a^	Dilmah	–	Bags
57.	Daily Superior	Irving	–	Loose
58.	Daily Classic	Irving	–	Bags
59.	Earl Grey^a^	Irving	–	Bags
60.	Russian Earl Grey	Lipton	–	Loose
61.	Yellow Label Tea^a^	Lipton	–	Loose
62.	Earl Grey Classic	Lipton	–	Bags
63.	Taste of London	Lipton	–	Bags
64.	Earl Grey^a^	Loyd Tea	–	Loose
65.	Fairtrade Luxury Gold Tea	Marks & Spancer	–	Bags
66.	Earl Grey^a^	Maraska	–	Loose
67.	Black Tea^a^	Minutka	–	Bags
68.	Black Tea^a^	Saga	–	Loose
69.	Black Tea^a^	Saga	–	Bags
70.	Earl Grey^a^	Saga	–	Bags
71.	English Breakfast^a^	Twinings	–	Loose
72.	Earl Grey^a^	Twinings	–	Loose
73.	Prince of Wales Tea	Twinings	–	Loose
74.	Earl Grey^a^	Twinings	–	Bags
75.	Simply Tea	Twinings	–	Bags

^a^Various producer or confections of tea the same name

### Preparation of Samples and Elemental Analysis

The bulk teas were homogenized and representative samples were mineralized in an electric furnace and then analyzed by flame atomic absorption spectrometry (FAAS) according to the previously published procedure by Brzezicha-Cirocka et al. [19, 20].

### Method Validation

The limit of detection (LOD) and limit of quantification (LOQ) for all the analyzed elements were calculated based on the independently prepared blank samples measurements. According to the method described by Konieczka and Namieśnik [21], LODs were set to blank means +3SD, where blank mean is a result of all blank samples measurements and SD is their standard deviation; whereas LOQ was calculated by multiplying LOD by a factor of three. Data for the validation procedure are given in Table 2.

**Table 2:** Results of the validation procedure of the analytical methodology

Element	Linearity			LOD (mg/100 g)	LOQ (mg/100 g)
	Calibration curve range (μg/mL)	Calibration curve	*R* ^2^		
Ca	2.00–15.0	*y* = 0.05801× + 0.0089	0.999	0.020	0.060
K	0.50–1.50	*y* = 0.00048× + 0.0149	0.997	0.040	0.120
Mg	0.10–0.90	*y* = 0.00107× + 0.0220	0.998	0.020	0.060
Na	0.50–1.20	*y* = 0.00071× + 0.0192	0.996	0.020	0.060
P	0.10–1.20	*y* = 0.00444× + 0.0117	0.999	0.030	0.090
Mn	0.15–5.00	*y* = 0.00015× + 0.0058	0.999	0.020	0.060
Fe	1.00–10.0	*y* = 0.00006× + 0.0082	0.996	0.010	0.030
Zn	0.20–1.50	*y* = 0.00034× + 0.0079	0.998	0.020	0.060
Cu	0.50–4.00	*y* = 0.00013× + 0.0022	0.999	0.009	0.027
Co	1.00–5.00	*y* = 0.00008× + 0.0052	0.999	0.003	0.009
Cd	0.20–2.00	*y* = 0.00035× + 0.0040	0.999	0.003	0.009
Cr	0.20–2.00	*y* = 0.00005× + 0.0007	0.999	0.001	0.003
Ni	0.50–2.00	*y* = 0.00008× + 0.0007	0.999	0.002	0.006
Pb	0.20–2.00	*y* = 0.00004× + 0.0004	0.999	0.004	0.012

The reliability of the method was determined using certified reference materials, i.e., Oriental Basma Tobacco Leaves (INCT-OBTL-5) and Polish Virginia Tobacco Leaves (INCT-PVTL-6). They were prepared according to the same procedure as the analytical samples. Recoveries of the studied elements ranged between 87 and 113 % (RSDs between 0.02–10.3 %) of the certified values for all the elements (Table 3).

**Table 3:** Element concentrations and RSD with recovery data for the certified reference materials Oriental Basma Tobacco Leaves (INCT-OBTL-5) and Polish Virginia Tobacco Leaves (INCT-PVTL-6)

Element	Certified values (mg/100 g)	Determined values (mg/100 g)	RSD (%)	Recovery (%)
Ca^a^	3859 ± 142	3566 ± 149	4.17	92
Ca^b^	2297 ± 78	2513 ± 13.4	0.50	109
Co^a^	0.10 ± 0.007	0.09 ± 0.005	5.65	90
Cu^a^	1.01 ± 0.04	1.00 ± 0.01	1.42	99
Cu^b^	0.51 ± 0.02	0.54 ± 0.01	2.00	106
Cd^a^	0.26 ± 0.01	0.28 ± 0.01	3.48	105
Cd^b^	0.22 ± 0.01	0.21 ± 0.001	0.40	95
Cr^a^	0.63^c^	0.56 ± 0.0001	0.02	89
Cr^b^	0.09^c^	0.09 ± 0.001	1.30	98
Mg^a^	853 ± 34	845 ± 4.96	0.59	99
Mg^b^	241 ± 9	247 ± 6.01	2.40	102
Mn^a^	18.0 ± 0.6	20.2 ± 0.34	1.67	112
Mn^b^	13.6 ± 0.5	15.3 ± 0.18	1.20	113
Zn^a^	5.24 ± 0.18	5.59 ± 0.24	4.31	107
Zn^b^	4.36 ± 0.1	4.64 ± 0.01	0.30	106
K^a^	2271 ± 76	2449 ± 50.3	2.06	108
K^b^	2640 ± 90	2692 ± 31.6	1.20	102
Na^b^	6.24^c^	5.50 ± 0.03	0.50	88
Pb^a^	0.20 ± 0.03	0.19 ± 0.01	5.60	95
Pb^b^	0.10 ± 0.01	0.09 ± 0.01	10.3	89
P^a^	170 ± 12	170 ± 0.41	0.24	100
P^b^	242 ± 5	239 ± 0.96	0.40	99
Ni^a^	0.85 ± 0.05	0.80 ± 0.078	9.80	94
Ni^b^	0.15 ± 0.01	0.13 ± 0.001	0.6	87
Fe^a^	149^c^	160 ± 1.01	0.63	107
Fe^b^	25.8^c^	28 ± 0.30	1.1	109

^a^Oriental Basma Tobacco Leaves INCT-OBTL-5

^b^Polish Virginia Tobacco Leaves INCT-PVTL-6

^c^Information value

## Statistical Analysis

The Shapiro-Wilk test showed that the data were not normally distributed; therefore, nonparametric tests were applied [22]. Moreover, data standardization was adopted, and the correlation analysis was performed using Spearman rank analysis. Kruskal-Wallis test, factor analysis, and cluster analysis were conducted in order to obtain statistically significant information about the quality and origin of samples.

## Results and Discussion

### Macroelements

The highest contents of Ca and Na were found in marketed teas (264 and 89.3 mg/100 g, respectively), whereas Indian and Kenyan teas were characterized by the lowest Na level (30.7 and 21.8 mg/100 g, respectively). Products from China, India, Ceylon, and Kenya had similar amounts of Ca (153–168 mg/100 g). Comparable Ca results (215 mg/100 g) were obtained by Dambiec et al. [23], whereas similar Na levels (88 mg/100 g) were reported by Soomro et al. [24] and Yemane et al. [25]. Among all macroelements, the highest levels were found for K (2349–2981 mg/100 g). Considerable variation was found in the content of Mg, as the highest levels amounted to 822 mg/100 g in Indian teas, and the lowest to 518 mg/100 g in marketed teas (Table 4). The latter value is higher than the one obtained by Gerbresadik and Chandravanshi [26] (354 mg/100 g). Indian, Ceylon, and Kenyan products contained similar amounts of P (305–359 mg/100 g), and Chinese tea had the highest P concentration (408 mg/100 g). Malik et al. [27] published comparable results for P (366 mg/100 g). Dambiec et al. [23] estimated a percentage of Na leaching (45.3 %) similar to ours (40.0 %), while Szymczycha-Madeja et al. [17] classified this macroelement as highly extractable (>55 %), which was confirmed in the case of teas from Kenya (62.0 %). The percentages of Ca leaching were the highest in Chinese teas (17.2 %) and the lowest in Kenyan teas (5.89 %). The lowest percentage of Mg leaching was found in Chinese teas (29.6 %), which is comparable to findings reported by Dambiec et al. [23]. Magnesium and P leaching percentages ranged between 29.6–39.7 and 26.8–38.2 %, respectively. The average percentage of Mg extraction to infusions (35.3 %) obtained by Dambiec et al. [23] and Gallaher et al. [28] is comparable to our results.

**Table 4:** Concentration of bioelements and toxic metals in dry tea samples in milligrams/100 g ($\overset{-}{x}$ ± SD range) and percentage of leaching

Elements	China	India	Ceylon	Kenya	Marketed
*n*	12 × 3	17 × 3	10 × 3	4 × 3	75 × 3
Ca	161 ± 101	153 ± 35	168 ± 51	155 ± 33	264 ± 52
	(22–449)	(105–220)	(87–250)	(105–193)	(133–420)
	17.2 ± 12.0 %	11.1 ± 8.22 %	9.54 ± 6.37 %	5.89 ± 1.60 %	11.1 ± 7.06 %
K	2666 ± 223	2803 ± 161	2981 ± 153	2738 ± 119	2349 ± 271
	(2322–2992)	(2474–3084)	(2656–3207)	(2536–2838)	(1845–3057)
	28.3 ± 8.03 %	33.6 ± 4.65 %	30.6 ± 6.25 %	28.4 ± 4.27 %	23.1 ± 4.08 %
Mg	764 ± 77	822 ± 103	769 ± 41.7	774 ± 63.1	518 ± 253
	(669–936)	(601–1052)	(704–829)	(699–873)	(164–901)
	29.6 ± 8.26 %	36.5 ± 3.07 %	35.6 ± 3.59 %	39.7 ± 4.37 %	35.1 ± 11.8 %
Na	67.3 ± 61.4	30.7 ± 11.5	55.5 ± 31.0	21.8 ± 6.06	89.3 ± 126
	(24.2–267)	(17.5–60.3)	(23.9–122)	(13.4–30.5)	(10.4–728)
	29.7 ± 11.4 %	47.8 ± 18.2 %	32.6 ± 12.2 %	62.0 ± 12.7 %	27.7 ± 20.7 %
Mn	30.9 ± 11.8	30.4 ± 10.5	27.7 ± 10.3	53.2 ± 12.6	56.3 ± 28.4
	(11.7–59.1)	(12.8–49.0)	(16.4–50.0)	(33.2–68.2)	(16.1–143)
	41.4 ± 14.4 %	44.8 ± 11.3 %	39.1 ± 8.53 %	44.3 ± 9.51 %	25.1 ± 7.00 %
P	408 ± 58.0	359 ± 39.9	305 ± 52.8	344 ± 25.3	296 ± 66
	(317–482)	(309–460)	(236–434)	(310–380)	(198–490)
	34.1 ± 9.50 %	34.7 ± 8.66 %	26.8 ± 8.53 %	31.6 ± 5.35 %	38.2 ± 7.66 %
Fe	0.90 ± 0.26	0.57 ± 0.29	0.43 ± 0.09	0.53 ± 0.01	0.73 ± 0.37
	(0.37–1.35)	(0.30–1.22)	(0.30–0.63)	(0.51–0.54)	(0.23–2.50)
	38.7 ± 17.9 %	38.0 ± 26.1 %	36.7 ± 22.0 %	29.1 ± 9.62 %	37.5 ± 23.8 %
Zn	4.25 ± 0.63	3.70 ± 0.60	2.70 ± 0.52	2.74 ± 0.22	3.29 ± 0.67
	(3.26–5.72)	(2.92–5.05)	(2.11–3.91)	(2.50–2.99)	(2.04–5.22)
	34.6 ± 11.2 %	36.2 ± 10.6 %	41.3 ± 10.6 %	45.1 ± 4.11 %	33.9 ± 11.3 %
Cu	2.25 ± 0.42	2.39 ± 0.50	1.83 ± 0.14	1.85 ± 0.17	2.28 ± 0.57
	(1.55–2.91)	(1.67–3.34)	(1.62–2.07)	(1.67–2.07)	(1.26–3.98)
	15.4 ± 3.42 %	16.9 ± 2.57 %	15.0 ± 1.75 %	14.1 ± 1.91 %	18.9 ± 5.34 %
Co	0.03 ± 0.01	0.02 ± 0.01	0.02 ± 0.01	0.02 ± 0.01	0.02 ± 0.01
	(0.01–0.04)	(0.01–0.04)	(0.01–0.05)	(0.01–0.04)	(0.01–0.05)
	39.4 ± 21.3 %	40.0 ± 25.1 %	43.6 ± 20.0 %	33.9 ± 4.41 %	33.4 ± 18.7 %
Cd	0.005 ± 0.001				
	(<LOD-0.007)	<LOD	<LOD	<LOD	<LOD
	<LOD				
Cr	0.08 ± 0.02	0.07 ± 0.03	0.05 ± 0.01	0.11 ± 0.06	0.17 ± 0.16
	(0.04–0.11)	(0.04–0.12)	(0.04–0.06)	(0.04–0.18)	(0.04–1.28)
	24.1 ± 8.48 %	34.1 ± 13.4 %	28.2 ± 9.34 %	31.7 ± 6.95 %	28.1 ± 13.3 %
Ni	0.50 ± 0.21	0.58 ± 0.13	0.35 ± 0.10	0.36 ± 0.05	0.42 ± 0.15
	(0.22–1.01)	(0.28–0.85)	(0.22–0.63)	(0.29–0.44)	(0.10–0.93)
	56.2 ± 18.6 %	67.7 ± 11.3 %	68.8 ± 17.2 %	75.8 ± 17.0 %	64.0 ± 24.4 %
Pb	0.05 ± 0.04	0.02 ± 0.01	0.02 ± 0.01	0.013 ± 0.003	0.03 ± 0.04
	(0.01–0.15)	(<LOD-0.06)	(0.01–0.04)	(0.01–0.02)	(<LOD-0.32)
	33.9 ± 21.3 %	18.9 ± 22.2 %	28.7 ± 28.6 %	45.4 ± 22.4 %	27.4 ± 26.4 %

LOD for Cd = 0.003 mg/100 g; LOD for Pb = 0.004 mg/100 g

*n* number of samples multiplied by number of analytical subsample

### Microelements

Manganese was the microelement found in the highest concentrations in our study, and the highest levels were determined in Kenyan and marketed teas (53.2 and 56.3 mg/100 g, respectively). Shaltout and Abd-Elkader [29] reported a slightly higher average Mn content (61.8 mg/100 g). Chinese samples had the highest Fe and Zn content (0.90 and 4.25 mg/100 g, respectively). According to Mupenzi et al. [30], low Fe concentration can be induced by high Mn levels. What is more, high Fe levels can cause Mn deficiency in tea plants. The determined amounts of Zn are comparable to those obtained by Al-Oud [31] for Chinese and Indian teas (2.67–5.39 mg/100 g). Copper levels in samples from Ceylon and Kenya (1.83 and 1.85 mg/100 g) were comparable and slightly lower than in Chinese, Indian, and marketed teas (2.25, 2.39, and 2.28 mg/100 g, respectively). Similar Cu results were reported by McKenzie et al. [32] (1.70 mg/100 g). Cobalt levels, which amounted to 0.02 mg/100 g, were comparable to those reported by Shaltout and Abd-Elkader [29]. Chinese and Indian samples had similar amounts of Cr (0.08 and 0.07 mg/100 g) and Ni (0.50 and 0.58 mg/100 g). Similar results were obtained by Shaltout and Abd-Elkader [29] for Ni (0.61 mg/100 g) and by Barone et al. [4] for Cr (0.04 mg/100 g). Barone et al. [4] reported in their study that Chinese tea samples generally had higher levels of elements than those originating from India.

The lowest percentage of leaching of Mn (25.1 %) was found in marketed teas and the highest (44.8 %) in Indian teas. For Fe and Zn, percentages of leaching showed similar levels in all the analyzed tea samples (29.1–38.7 and 33.9–45.1 %, respectively). Among all the analyzed elements, Ni showed the highest percentage of leaching, which ranged from 56.2 % (Chinese tea) to 75.8 % (Kenyan tea).

Many factors influence the contents of trace metals in tea leaves. According to Milani et al. [33], variations in mineral composition of tea leaves can be explained by the age of leaves used in the production (old or young), soil composition, rainfall amount, and growing conditions in general. Unfortunately, commercial teas often have unknown geographical origin, as they are a mixture of leaves from different locations [33]. Black tea is produced through leaf fermentation in contrast to green tea, which results in higher levels of certain trace elements [34]. Regulations have been established for many vegetable products in many countries, but the European Union has no specified regulations about the acceptable metal content in tea.

### Toxic Metals

In general, the analyzed samples contained more Pb than Cd. The levels of Cd varied little, i.e., 0.003–0.005 mg/100 g. Similar results for Cd were obtained by Milani et al. [33] (0.001–0.002 mg/100 g) and significantly lower ones by Barone et al. [4] (0.0004 mg Cd/100 g). Chinese teas had the highest Pb content (0.05 mg/100 g), which is comparable with the results reported by Barone et al. [4] (0.05 mg Pb/100 g). The percentage of leaching of Pb to infusions varied considerably, as the highest was found for Kenyan teas (45.4 %) and the lowest for Indian ones (18.9 %).

The contents of heavy metals in tea leaves may be a result of contamination that can be caused by many factors such as use of various manufacturing and agronomic processes and of fertilizers [29, 35, 36]. Among the main sources of Pb in the environment are leaded fuel, waste incineration, and industry [37]. Lead pollution is correlated with urbanization and population density. Moreover, higher levels of this heavy metal in tea samples can be attributed to contamination during the process of tea production and its packing [37]. Cadmium found in tea leaves might be a result of phosphate and zinc fertilizers usage [38]. It is estimated that Cd from phosphate fertilizers constitutes >50 % of the total input to agricultural land not heavily polluted or heavily industrialized [39, 40]. Sarma et al. [41] reported that heavy metal contaminations of tea leaves might be explained by the position of the tea cultivation area. In that study, an Assam plantation was situated in the vicinity of the oldest crude oil exploration station, and accidental spillage during drilling and transportation could be the main sources of tea field contamination. Moreover, it has been established that heavy metals enter into plant bodies in acidic soil, in which tea usually grows [42].

### Correlation

Nonparametric Spearman’s rank test was performed at three levels of significance (*p* < 0.05, *p* < 0.01, *p* < 0.001), and both positive and negative correlations were found between the analyzed elements. The most significant positive correlations (*p* < 0.001) were found for P-Zn, Mn-Co, and Mn-Cr for all the countries. There were also recorded important interelement correlations among black tea samples from Indian and Ceylon plantations. Strong interdependences (*p* < 0.01) were also found between Ca-Mn, P-Zn, Mn-Co, Zn-Ni, and Cu-Co.

### Kruskal-Wallis Test

Through the Kruskal-Wallis test, it was possible to determine statistically significant differences in the analyzed database. Relationships were found between the geographical provenance of tea and concentrations of elements including the following: Na (*H* = 18.596; *p* = 0.001), P (*H* = 14.533; *p* = 0.001), Mn (*H* = 9388; *p* = 0.025), Fe (*H* = 12.002; *p* = 0.007), Zn (*H* = 26.166; *p* = 0.000), Cu (*H* = 12.861; *p* = 0.005), and Ni (*H* = 11.929; *p* = 0.008). Dunn’s test was also performed, confirming the outcome of the Kruskal-Wallis test. The results of Dunn’s test are shown in Table 5. Kruskal-Wallis test and Dunn’s test (Table 6) were also performed for tea samples from Asia, i.e., India (plantation Assam and Darjeeling) and the island of Ceylon. Relationships were found between provenance of tea and concentrations of several elements: Na (*H* = 10.739; *p* = 0.005), P (*H* = 10.992; *p* = 0.004), Mn (*H* = 7.218; *p* = 0.027), Fe (*H* = 11.833; *p* = 0.003), Zn (*H* = 14.679; *p* = 0.001), Cu (*H* = 13.443; *p* = 0.001), Cr (*H* = 7.382; *p* = 0.025) and Ni (*H* = 12.232; *p* = 0.002).

**Table 5:** Results of the post hoc Dunn’s test conducted for the analyzed data matrix for tea samples from Ceylon, China, India, and Kenya. There are only given elements for which *p* < 0.05

	Ceylon	China	India	Kenya
Ceylon	–	P, Zn, Fe	Cu, Na, Ni, Zn	Na
China	P, Zn, Fe	–	Na, Fe	Na, Zn
India	Cu, Na, Ni, Zn	Na, Fe	–	
Kenya	Na	Na, Zn		–

**Table 6:** Results of the post hoc Dunn’s test conducted for the analyzed data matrix for tea samples from India and Ceylon. There are only given elements for which *p* < 0.05

	Ceylon	Assam	Darjeeling
Ceylon	–	Na, Cu, Cr, Ni	P, Fe, Zn, Ni
Assam	Na, Cu, Cr, Ni	–	Mn, Fe
Darjeeling	P, Fe, Zn, Ni	Mn, Fe	–

### Factor Analysis

**Fig. 1:**
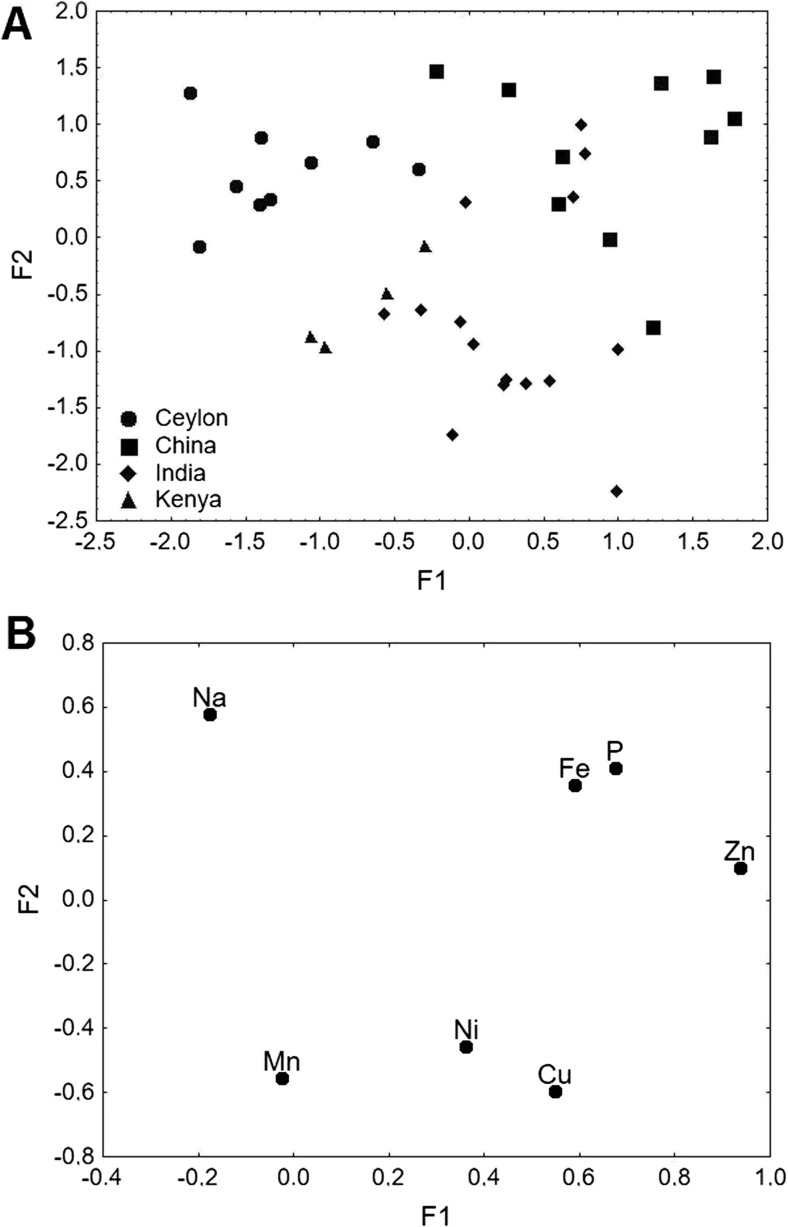
**a** Scatter plot of objects samples of two factors of the all tea samples from Ceylon, China, India, and Kenya. **b** Scatter plot of loading for elements in all the analyzed tea samples from Ceylon, China, India, and Kenya

The results of the factor analysis (FA), conducted on raw data sets of black tea obtained from tea shops, are shown in Fig. 1a and b. Factor analysis performed with all the analyzed metals did not give clear output, and thus, we decided to narrow the data set to the elements Na, Mn, Ni, Cu, Fe, P, and Zn. Factor analysis was applied to data of elements which were found significant in the Kruskal-Wallis test. The final choice of descriptors was done by a series of factor analyses, which were performed in order to verify the clarity of the outcome. As a result, two factors were obtained, i.e., F1 (35.7 % of the total variance) and F2 (23.5 % of the total variance). Both factors cumulatively explain 59.2 % of the total variance, whereas the eigenvalues for F1 and F2 are 2.14 and 1.41, respectively. As can be seen in Fig. 1a, samples from all the analyzed regions can be distinguished, i.e., Ceylon, China, India, and Kenya. The scatter plot of loadings was drawn for F1–F2 in order to identify elements responsible for the grouping of objects (Fig. 1b). Higher values of F1 correspond to Indian and Chinese samples, which were described by Ni, Cu, Fe, P, and Zn. Phosphorus, Fe, and Zn were responsible for differentiation of Chinese tea, whereas the highest amounts of Ni and Cu were noted in Indian samples. Such high levels of these metals in samples of these two origins could possibly be explained by product contamination during its manufacture or area pollution. Simultaneously, high amounts of P in Chinese samples may be related to the increasing use of fertilizers, which was confirmed by research conducted by Mupenzi et al. [30].

Lower values of F1 characterize Kenyan and Ceylon tea samples which are described by Mn and Na (Fig. 1a, b). Kenyan tea was differentiated by Mn as significant differences were found in Mn concentrations between these two groups of tea. Sodium was found to be a descriptor of Ceylon teas, which may be associated with geographical location of the plantation, as Ceylon is an island surrounded by the waters of the Indian Ocean. Higher values of F2 described Ceylon, Chinese, and partly Indian tea samples, which can be identified by Na, Fe, P, and Zn. Lower values of F2 were associated with Indian and Kenyan samples, which were differentiated by Mn, Ni, and Cu (Fig. 1a, b).

**Fig. 2:**
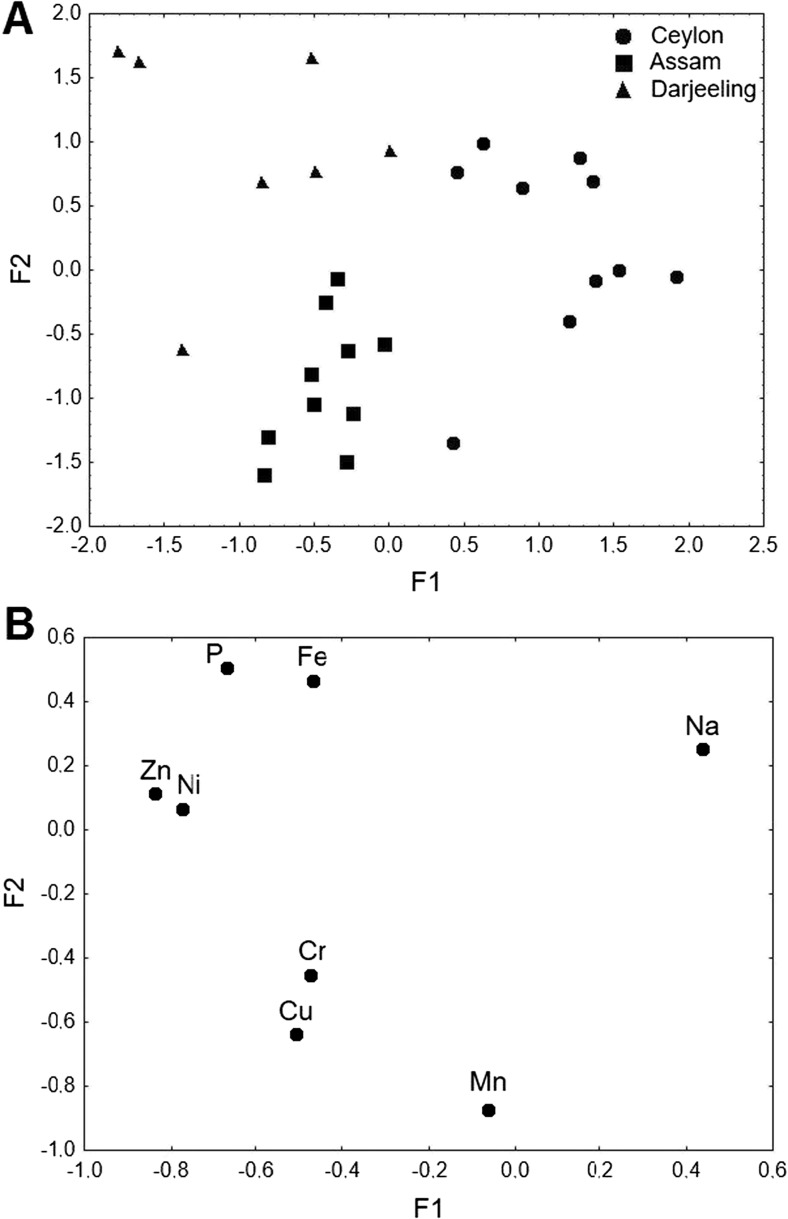
**a** Scatter plot of objects samples of two factors of the all tea samples from India plantations and Ceylon. **b** Scatter plot of loadings for elements in all the analyzed tea samples from India plantations and Ceylon

Factor analysis was also performed for Indian plantations, i.e., Assam and Darjeeling, and Ceylon samples in order to differentiate samples within plantations (Fig. 2a, b). It was found that 57.2 % of the total variance is explained by F1 (33.1 %) and F2 (24.1 %). The eigenvalues were 2.64 for F1 and 1.93 for F2. The factor analysis clearly differentiated between tea samples originating from different plantations. Ceylon samples were described by higher F1 values and Na, which might be associated with its significant content in soils of this country (Fig. 2a, b). Lower values of F1 corresponded to Assam and Darjeeling samples, which were described by P, Fe, Zn, Ni, Cr, Cu, and Mn. Tea samples from Darjeeling were differentiated by P, Fe, and Zn, whereas Assam tea samples were significantly correlated with Cr, Cu, and Mn. Factor 2 differentiated samples from India (Assam and Darjeeling) as its higher values corresponded to Darjeeling plantations and lower values to Assam ones. Although Ni is characterized with positive F2 loading, there were no significant variations in its content in Assam and Darjeeling samples.

### Cluster Analysis

**Fig. 3:**
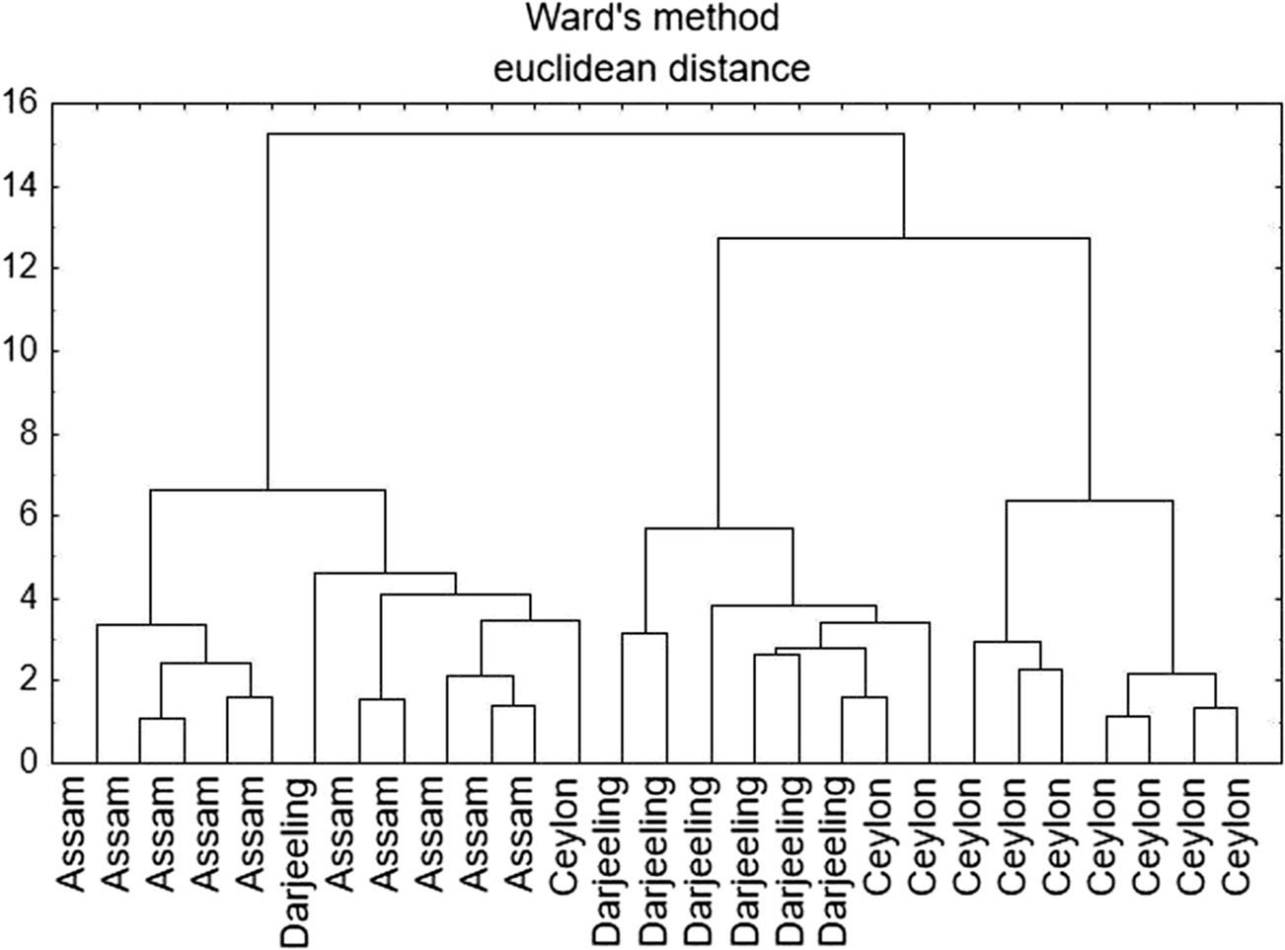
Hierarchical dendrogram for tea samples from India and Ceylon plantations

The cluster analysis (CA) was based on Ward’s method with the usage of the Euclidean distance. Application of CA made it possible to differentiate samples according to their origin, i.e., Ceylon and Indian plantations (Fig. 3). Assam plantations were discriminated by Cr, Cu, and Mn, and Darjeeling ones by Fe, Zn, and P. Ceylon tea samples corresponded to Na, which was already confirmed to be a discriminative element for this region. However, only few samples were assigned to the improper cluster, which might be due to the similarity between samples, especially in view of their contamination with heavy metals. What is more interesting, it was noted that Darjeeling samples are more similar to Ceylon samples than to the other Indian plantation (Assam) (Fig. 3).

Thus, it can be concluded that CA similarly to FA is able to distinguish tea samples in view of their geographical origin, which might be helpful when authenticity assessment and fraud detection is necessary.

### Recommended Dietary Intake

The daily intake of bioelements from tea was evaluated in view of the latest available Polish [43] and American recommended dietary intakes (RDA) [44]. Consumption of one cup of black tea (200 mL) results in intakes of Ca, K, Na, Mg, and P in the range of 0.02–1.30 % of their respective RDAs. Thus, black tea is not a rich source of these macroelements, despite the fact that their concentrations in tea leaves are the highest among all the analyzed elements (Table 7). Realization of the recommended daily intakes for microelements such as Fe, Zn, Cu, and Ni is less than 1.0 %. For Cr, intake of 200 mL of tea infusion supplies 2.8 and 4.0 % of the RDA for men and women, respectively. Black tea is a significant source of Mn and Co, one cup daily provides 15 % of the RDA for Mn and 10 % for Co. However, Mn bioavailability amounts up to 40 % [45]. Therefore, only 6 % of Mn present in one cup of black tea will be absorbed. Moreover, according to the World Health Organization [46] there is no quantitative information available to indicate toxic levels of manganese in the human diet. High Co content in tea leaves may possibly be explained by the use of Co-containing fertilizers. There is evidence that Co in higher plants promotes the formation of chlorophyll and plant growth [47]. According to Kabata-Pendias and Szteke [47], approximately 50 % of Co will be absorbed in the gastrointestinal tract. Furthermore, Co absorption can be increased among individuals who are Fe deficient.

**Table 7:** Comparison of Recommended Dietary Allowance (for a person weighing 70 kg through consumption of one cup (200 mL) of black tea beverage) to the daily intake from tea with consideration of PTWI for Pb

Element	Recommended daily allowance (RDA) (mg/day/person)		Average content in 200 mL of infusion (mg/200 mL)	Percentage of RDA	
	Male	Female		Male	Female
	(31–50 years)	(31–50 years)		(31–50 years)	(31–50 years)
Ca	1000	1000	0.49 ± 0.38	0.05	0.05
			0.03–1.83		
K	4700	4700	13.2 ± 4.27	0.3	0.3
			4.19–24.5		
Mg	420	320	4.21 ± 1.99	1.0	1.3
			0.46–8.34		
Na	1500	1500	0.27 ± 0.12	0.02	0.02
			0.07–0.67		
Mn^a^	2.3	1.8	0.27 ± 0.13	11.7	15
			0.07–0.84		
Fe^b^	10	18	0.005 ± 0.003	0.05	0.03
			0.0002–0.01		
P	700	700	2.29 ± 0.69	0.3	0.3
			0.98–4.54		
Zn	11	8	0.02 ± 0.009	0.2	0.2
			0.001–0.056		
Cu	0.9	0.9	0.01 ± 0.003	1.1	1.1
			0.003–0.017		
Co^c^	0.002	0.002	0.0002 ± 0.0001	10	10
			<LOD-0.0005		
Cr^a^	0.035	0.025	0.001 ± 0.0004	2.8	4.0
			0.00005–0.002		
Ni^a^	1	1	0.006 ± 0.003	0.6	0.6
			<LOD-0.01		
Element	PTWI	PTWI for a person weighing 70 kg	The average content in 200 mL beverage (mg/200 mL)	Realization of PTWI through consumption of one cup daily per week of 200 mL of the product for a person weighing 70 kg (%)	
Pb	25 μg/kg	1750	0.0002 ± 0.0001	0.01	
			<LOD-0.0007		

LOD for Co = 0.0001 mg/200 mL; LOD for Ni = 0.00004 mg/200 mL; LOD for Pb = 0.0001 mg/200 mL

^a^American recommendations [44]

^b^Polish recommendations [43]

^c^In the form of vitamin B_12_

There were also assessed levels of heavy metals such as Pb (Table 7) and Cd in the infusions, but the latter was under the limit of detection of the method applied. Thus, there could not have been estimated its provisional tolerable weekly intake (PTWI) realization. Former PTWI dose for Pb as recommended by the WHO/FAO [48] should not exceed 25 μg/kg, but it was withdrawn by the 73rd report of the Joint FAO/WHO Expert Committee of Food Additives [49]. It was found that it is not possible to establish a new dose PTWI that would be health protective. The PTWI’s analyses were based on the earlier guidelines of WHO/FAO [48] for a person weighing 70 kg. The average Pb levels in 200 mL beverage amounted to 0.0002 mg. Therefore, the consumption of one cup daily per week of 200 mL tea results in the realization of PTWI for Pb in 0.01 %. It can be concluded that drinking black tea does not result in exceeding PTWIs; thus, it poses no health risk for human.

## Conclusions

Although specified regulations concerning tea quality are not established in the European Union, it is important to control it as this beverage is one of the most commonly consumed. Therefore, we determined the elemental composition of various black tea samples originating from China, India, Ceylon, and Kenya. We conclude that there is no significant health risk associated with consumption of the analyzed tea samples, but that tea can constitute a valuable source of manganese in the human diet. Based on the obtained elemental data, the percentage of leaching as well as daily intake realization were assessed. The highest level of RDAs’ realization was noted for Mn (15 %) and Co (10 %). Verification of the interdependences between elemental composition and country of origin of samples was done by multivariate techniques such as factor analysis and cluster analysis. They allowed on differentiation of teas according to the country of origin, i.e., China, India, Ceylon, and Kenya. Moreover, they were found helpful in diversification of teas originating from various plantations within a single country. Thus, they proved to be good tools able to differentiate samples in view of their provenance as well as plantation within the common region.
